# Impaired layer specific retinal vascular reactivity among diabetic subjects

**DOI:** 10.1371/journal.pone.0233871

**Published:** 2020-09-11

**Authors:** Maxwell Singer, Bright S. Ashimatey, Xiao Zhou, Zhongdi Chu, Ruikang Wang, Amir H. Kashani

**Affiliations:** 1 Department of Ophthalmology, USC Roski Eye Institute, Keck School of Medicine of the University of Southern California, Los Angeles, California, United States of America; 2 Department of Bioengineering, University of Washington, Seattle, Washington, United States of America; 3 Department of Ophthalmology, University of Washington, Seattle, Washington, United States of America; 4 USC Ginsburg Institute for Biomedical Therapeutics, Keck School of Medicine of the University of Southern California, Los Angeles, California, United States of America; Boston Medical Center, Boston University School of Medicine, UNITED STATES

## Abstract

**Purpose:**

To investigate layer specific retinal vascular reactivity (RVR) in capillaries of diabetic subjects without DR or with only mild non-proliferative diabetic retinopathy (NPDR).

**Methods:**

A previously described nonrebreathing apparatus was used to deliver room air, 5% CO_2_, or 100% O_2_ to 41 controls and 22 diabetic subjects (with mild or no NPDR) while simultaneously acquiring fovea-centered 3x3mm^2^ Swept-Source Optical Coherence Tomography Angiography (SS-OCTA) images. Vessel skeleton density (VSD) and vessel diameter index (VDI) were calculated for each gas condition for the superficial retinal layer (SRL) and deep retinal layer (DRL). The superficial layer analysis excluded arterioles and venules. Data analysis was performed using mixed factorial analysis of covariance stratified by diabetic status. All models were adjusted for age, gender, and hypertension, and statistical significance for multiple comparisons from posthoc comparisons were defined at p<0.017.

**Results:**

Among controls, there was a significant difference in capillary VSD between all gas conditions (p<0.001). This difference was present in both the SRL and DRL. Among diabetics, there was no significant difference in response to CO_2_ conditions in the SRL (p = 0.072), and a blunted response to both CO_2_ (p = 0.9) and O_2_ in the DRL (p = 0.019). A significant gas effect was detected in the capillary VDI in the SRL of controls (p = 0.001), which was driven by higher VDI in the oxygen condition compared to that of carbon dioxide.

**Conclusions:**

Impairment in RVR in diabetic subjects is characterized by a paradoxical response to CO_2_ in both the SRL and DRL as well as an attenuated response to O_2_ in the DRL. These layer and gas specific impairments in diabetics seem to occur early in the disease and to be driven primarily at the capillary level.

## Introduction

Blood flow through the retinal vasculature changes according to the metabolic needs of local tissue and the presence or absence of metabolites in the local environment [1]. One example of this spatial and temporal heterogeneity in blood flow is the reactivity of retinal vessels to blood gas perturbations. Retinal vessels change in caliber, dilating in hypercapnic conditions and constricting in hyperoxic conditions [2–7]. Such changes are mediated by regulatory mechanisms that allow for decentralized regulation of blood flow within the larger vessels of the retina [8]. For example, retinal arteries and arterioles can change in diameter in response to changes in metabolic demand or supply [9–11]. This retinal vascular reactivity (RVR) has been shown to be blunted in diabetics [12–16] even in the absence of clinically apparent diabetic retinopathy (DR) [2]. Most studies have demonstrated reactivity changes in arterioles or venules, but few have demonstrated impairment at the capillary level [1, 6]. Unlike many other imaging modalities that have been used to assess retinal vascular reactivity at the capillary level, Optical Coherence Tomography Angiography (OCTA) is an FDA approved technology that is commercially available [17, 18]. Our group has recently used Swept-Source OCTA (SS-OCTA) to investigate the impact of room air, hypercapnia and hyperoxia conditions on retinal capillaries in full thickness retina between controls and subjects with DR. That study concluded that subjects with DR preferentially responded to hyperoxia but not hypercapnia, while the healthy controls responded to both [19].

There is currently growing evidence that the deep retinal vascular plexus is more severely damaged by diabetes than the superficial plexus. Even in the absence of clinically detectible retinopathy, the deep vascular plexus shows lower capillary density than in controls [20–24]. Given that vascular damage may occur early in disease and vary by retinal layer, we expand our previous studies by investigating layer specific retinal vascular reactivity between healthy controls and subjects with mild non-proliferative diabetic retinopathy (NPDR) or no DR.

## Methods

This was a prospective, observational study of diabetic patients and healthy controls. This study was approved by the institutional review board of the University of Southern California (USC) and adhered to the tenets of the Declaration of Helsinki.

### Subjects

Subjects were recruited at the USC Roski Eye Institute and informed consents were obtained from each subject. Subjects were excluded if they had any past ocular history of glaucoma or retinal surgery, or past medical history of vascular diseases including heart disease, surgery, or vasculitis. Only those diabetic subjects without clinically apparent DR or with only mild NPDR were included. All subjects with significant media opacity such as cataracts or any other obscuration of the fundus were excluded. Subjects who reported a history of syncope, shortness of breath, lung disease, congestive heart failure, or recent hospitalization of greater than 1-day duration within the last 12 months were also excluded to minimize risk of adverse events during gas breathing. All eyes were imaged at the USC Roski Eye Institute between October 2017 and February 2020. Approximately, 46% of controls and 36% of diabetics overlapped with those from our previous study by Ashimatey et al. [19]

### Gas delivery system

In order to create conditions of physiological hyperoxia and hypercarbia, a nonrebreathing apparatus was used to deliver gas mixtures. This gas delivery system has been described by Ashimatey et al., [19] and a step by step visual-illustration of the procedure is demonstrated in Kushner-Lenhoff et al. [25] In brief, three gas mixtures were delivered through a non-rebreathing gas apparatus to the subjects in the following order during the same visit: room air, a mixture of 5% carbon dioxide, atmospheric oxygen and balanced nitrogen, and finally 100% oxygen. There was a 10-minute refractory period between the hypercapnic and hyperoxic conditions to allow washout of the previous gas condition. The order of gas administration was established to maximize testing efficiency and to mitigate the impact of a persistent effect of a prior testing condition on the next condition. Room air was performed first because that was the baseline measure and acquiring images under room air condition after any gas breathing introduces the possibility of persistent gas effect from the prior gas condition. Yezhuvath et al. found that end tidal CO_2_ and cerebrovascular reactivity returned to baseline 60 seconds after 5% CO_2_ breathing and Tayyari et al suggested that an after-gas effect from O_2_ could persist longer that of CO_2_ [3, 26]. A 10-mins wash out time between CO_2_ and O_2_ was deemed adequate based on previous studies [3]. Therefore the order of CO_2_ and O_2_ gas administration was fixed for all subjects. During delivery of the gases, a fingertip pulse oximeter was used to monitor oxygen saturation. Testing was stopped if the oxygen saturation fell below 94% for any subject at any time.

### SS-OCTA imaging protocol

During gas breathing, images were acquired using a 100kHz SS-OCTA system (PLEX Elite 9000, software version 1.7; Carl Zeiss Meditec, Dublin, CA, USA). The light source has a central wavelength of 1060 nm and a bandwidth of 100 nm, providing an axial resolution of ~ 6μm and lateral resolution of ~ 20μm (estimated at the surface of retina). The device uses an optical microangiopathy (OMAG) algorithm [27, 28] to determine the motion signal, which is interpreted as representing the flow of erythrocytes. Three OCTA volumes (3x3mm field-of-view) centered on the fovea were acquired starting 60 seconds after the onset of each gas breathing condition. Before OCTA image acquisitions were started, the subjects were asked to keep their eyes closed immediately after the imaging set-up was completed and the retinal region to be imaged was focused and aligned. Subjects were asked to blink two times before any OCTA image acquisitions were initiated to enhance tear film stability. Artificial tears were administered between gas conditions and at the beginning of the imaging session to maximize image quality and signal strength.

Each OCTA volume is formed by 300 horizontal scans (B-scans) with each B-scan formed by 300 A-scans of 1536 pixels. One eye per patient was imaged, based on fixation ability, absence of co-morbid disease and media opacity. If both eyes were suitable, the imaged eye was chosen randomly. Images with poor decentration of the foveal avascular zone (FAZ), signal strength less than seven, significant motion artifacts or vitreous floaters were excluded. One representative image, the image with the highest signal strength and fewest artifacts, was selected per condition. The volumes were then automatically segmented into *en face* superficial retinal layer (SRL) and deep retinal layer (DRL). The segmentation software used is commercially available on the OCTA device and has been previously described by our group in the past [29, 30]. The SRL extends from the inner limiting membrane (ILM) to the inner plexiform layer (IPL), which is approximated as 70% of the thickness between the ILM and outer plexiform layer (OPL). The DRL extends from the IPL to the OPL, which is estimated to be 110μm above the retinal pigment epithelium (RPE). The *en face* DRL for the best images were projection-resolved using the commercially available projection artifact removal feature on the device [31, 32].

### Quantitative vessel morphometric measures

Quantitative analysis with custom MATLAB software was performed on SRL and DRL images to calculate measures that have been described previously [33]. Vessel area density (VAD) represents the total area of the image occupied by vessels and is calculated by dividing the area occupied by vessel pixels by the dimensions of the image after image binarization. Vessel skeleton density (VSD) represents the total length of the blood vessels. VSD is calculated by skeletonizing the diameter of each vessel to one pixel and then dividing the pixels that represent all the skeletonized vessels by the total area of the image. Vessel diameter index represents the caliber of the blood vessels and is calculated by dividing VAD by VSD [25, 29, 34].

**Fig 1:**
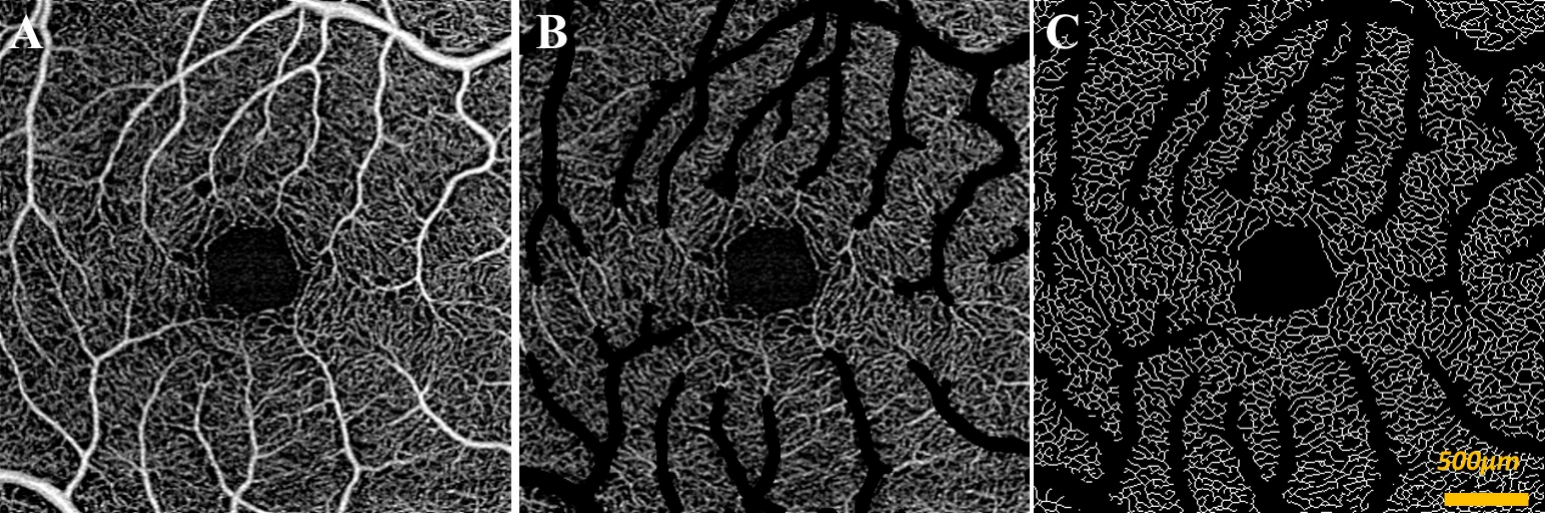
Large vessel exclusion of en face superficial retinal layer (SRL) 3x3mm SS-OCTA of control subject acquired during room air condition. (A) original image (B) original image with large vessels removed (C) image after large vessels excluded and remaining capillaries skeletonized.

The central 3x3mm foveal centered region of the macula contains small arterioles which may preferentially drive the RVR response. In order to examine the isolated effect of gas conditions on capillaries only, a custom semi-automated MATLAB software was developed specifically to exclude non-capillary vessels that occur in the SRL (Fig 1). A manual threshold was empirically determined to exclude arterioles and venules without excluding precapillary arteriolar segments. VSD is then calculated by dividing the remaining skeletonized capillaries by all the pixels of the image that were not occupied by large vessels. In addition, VSD, VDI, and VAD of the SRL with the large vessels included was also quantified for comparison. Unless otherwise specified, results presented are those with large vessels excluded.

### Statistical analysis

A 2 (layers: DRL, SRL) x 3 (gas conditions: room air, oxygen, carbon dioxide) mixed factorial analysis of covariance (ANCOVA) model with repeated effects of layer and breathing condition was used to assess differences in the OCTA metrics (VSD, VAD and VDI) by layer, gas breathing condition, and their interaction. Due to limitations in sample size and power to detect a three-way layer-gas-diabetes interaction, separate models were run for diabetic versus normal patients and for VSD measurements obtained for the images. All models were adjusted for age, gender, and hypertension. Differences of the differences in VSD by gas breathing condition between layers were estimated from the models. All statistical tests were two-sided with an alpha level set at 0.05 for statistical significance. The assumption of sphericity was assessed using the Mauchly’s assessment of sphericity, and when the assumption of sphericity was violated, the p-value associated with the degree of freedom adjustment using the Greenhouse-Geisser correction was reported. The Bonferroni method was applied for posthoc multiple comparisons at p = 0.017. All statistical analyses were performed using SPSS version 25 (IBM SPSS Statistics for Windows, Version 25.0. Armonk, NY).

## Results

**Fig 2:**
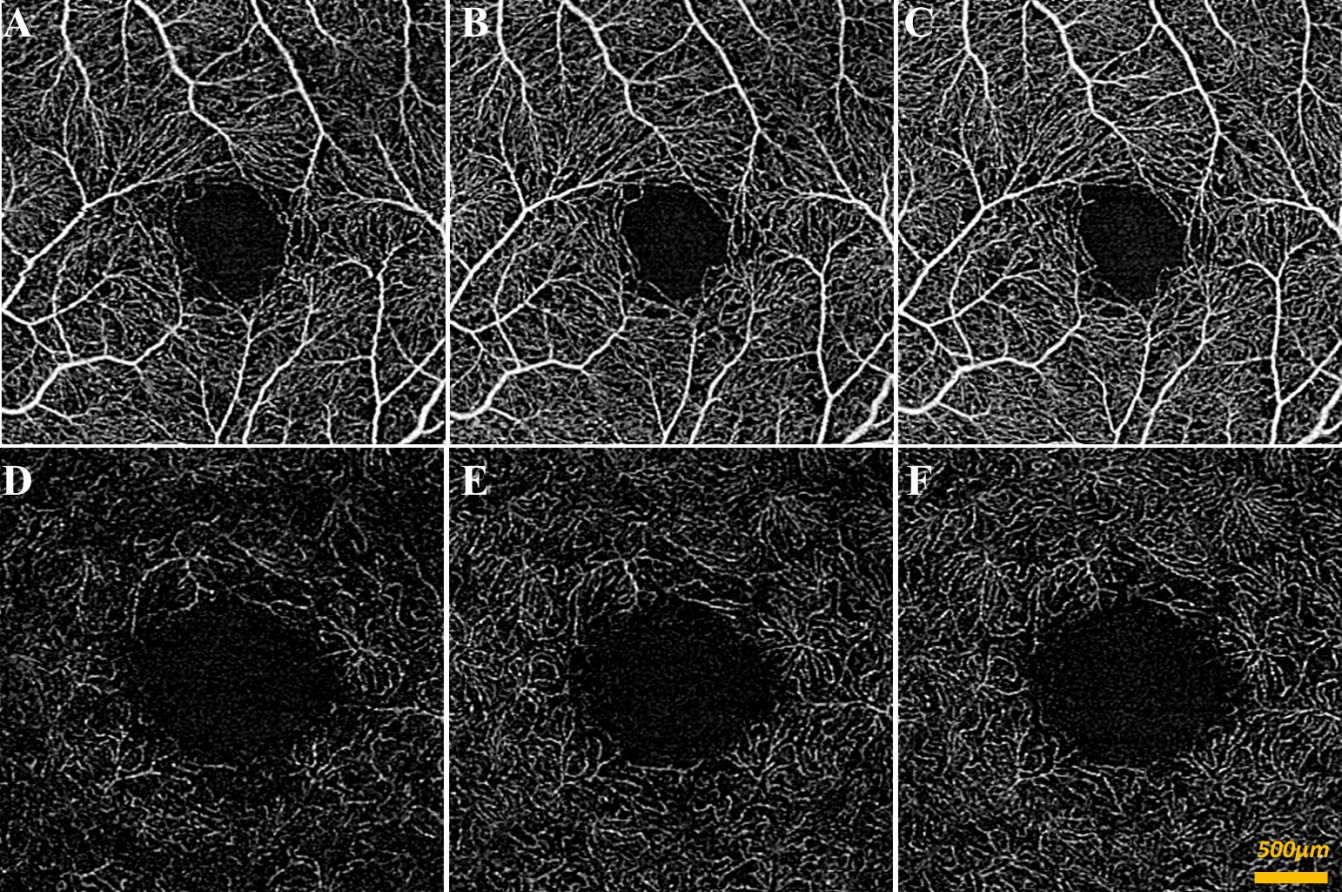
SS-OCTA of control subject during different gas conditions. (A-C) 3x3mm en face superficial retinal layer image of blood flow around central macula during oxygen condition (A), room air condition (B), and carbon dioxide condition (C). (D-E) 3x3mm en face deep retinal layer image of blood flow around central macula during oxygen condition (D), room air condition (E), and carbon dioxide condition (F). A qualitative increase in signal was apparent in the hypercapnic condition when compared to the other two conditions.

**Fig 3:**
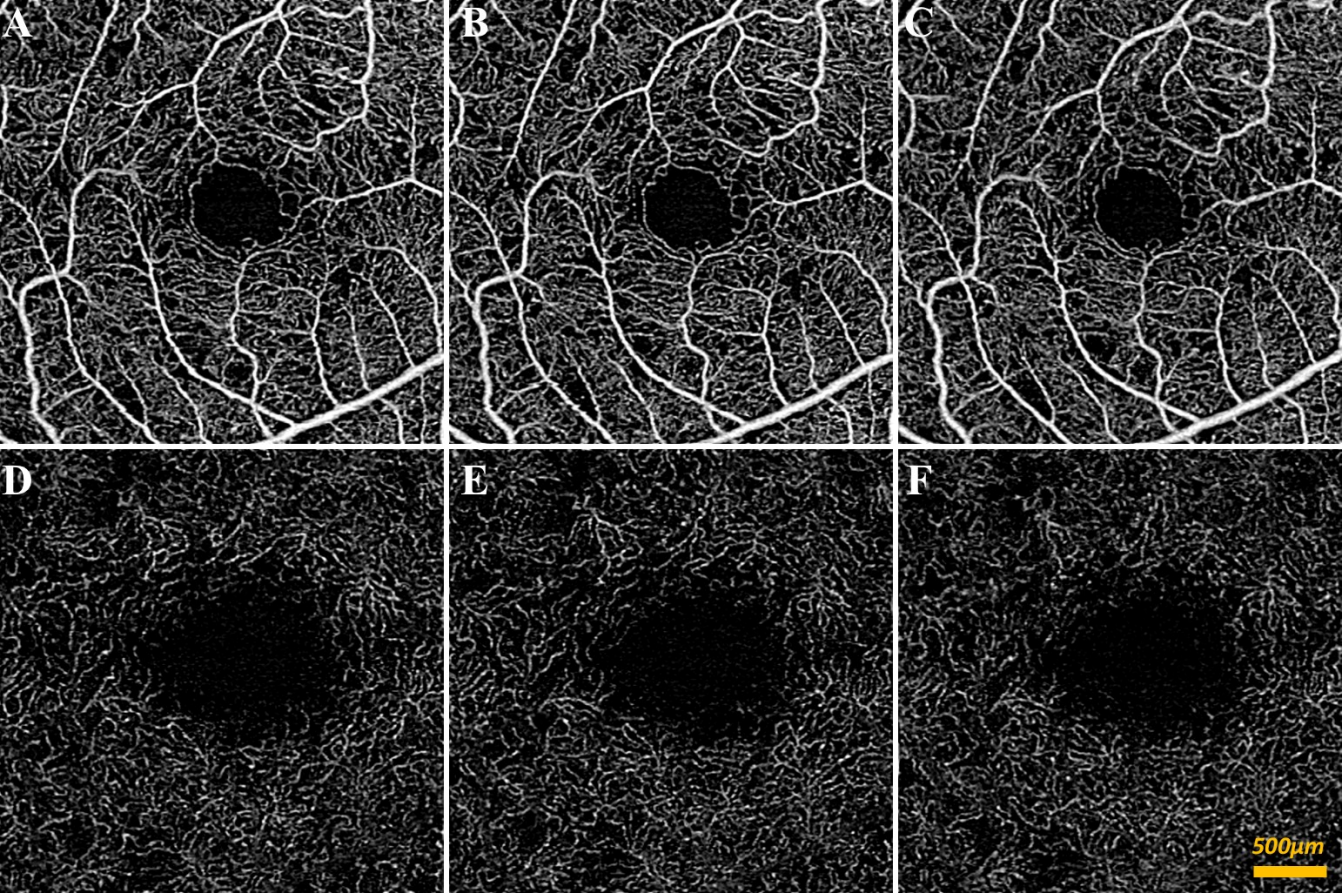
SS-OCTA of diabetic subject without diabetic retinopathy during different gas conditions. (A-C) 3x3mm en face superficial retinal layer image of blood flow around central macula during oxygen condition (A), room air condition (B), and carbon dioxide condition (C). (D-E) 3x3mm en face deep retinal layer image of blood flow around central macula during oxygen condition (D), room air condition (E), and carbon dioxide condition (F).

Our study included 41 control (mean age 53.0 ± 18.9) and 22 diabetic subjects (mean age 53.7 ± 16.7). Table 1 summarizes the subject demographics. Fig 2 shows qualitative representations of the decorrelation signal intensity and capillary density under different gas breathing conditions in the SRL and DRL for a control subject. The same representations are shown for a diabetic subject in Fig 3. Subtle signal intensity changes and the apparent density of capillaries in the control subject were qualitatively increased in the hypercapnic condition compared to the other two conditions. Quantitative analysis confirmed this finding by showing that significant effects on VSD of gas breathing condition, layer, and the gas-layer interactions were present for both controls and diabetics, after controlling for age, gender, and hypertension (all p<0.001). Below we elaborate on these findings in more detail.

**Table 1:** Subject demographics.

	Disease Classification	
	Controls	Diabetics
**Number (Subjects)**	41	22
**Age**	53.0 ± 18.9	53.7 ± 16.7
**Female gender**	20 (48.8%)	9 (40.9%)
**Hypertension**	10 (24.4%)	6 (27.3%)
**Severity of DR**	N/A	15 no DR, 7 mild NPDR
	**OCTA signal strength (mean, [Min, Max]) under gas non-rebreathing conditions**	
**RA**	9.8 [9,10]	9.8 [9,10]
**CO**_**2**_	9.7 [8,10]	9.9 [9,10]
**O**_**2**_	9.6 [8,10]	9.6 [9,10]

The number, age, gender, hypertension status, and ocular diagnosis of subjects is summarized. RA means Room air.

### Retinal vascular reactivity to CO_2_ is absent in SRL and DRL of diabetic subjects

**Fig 4:**
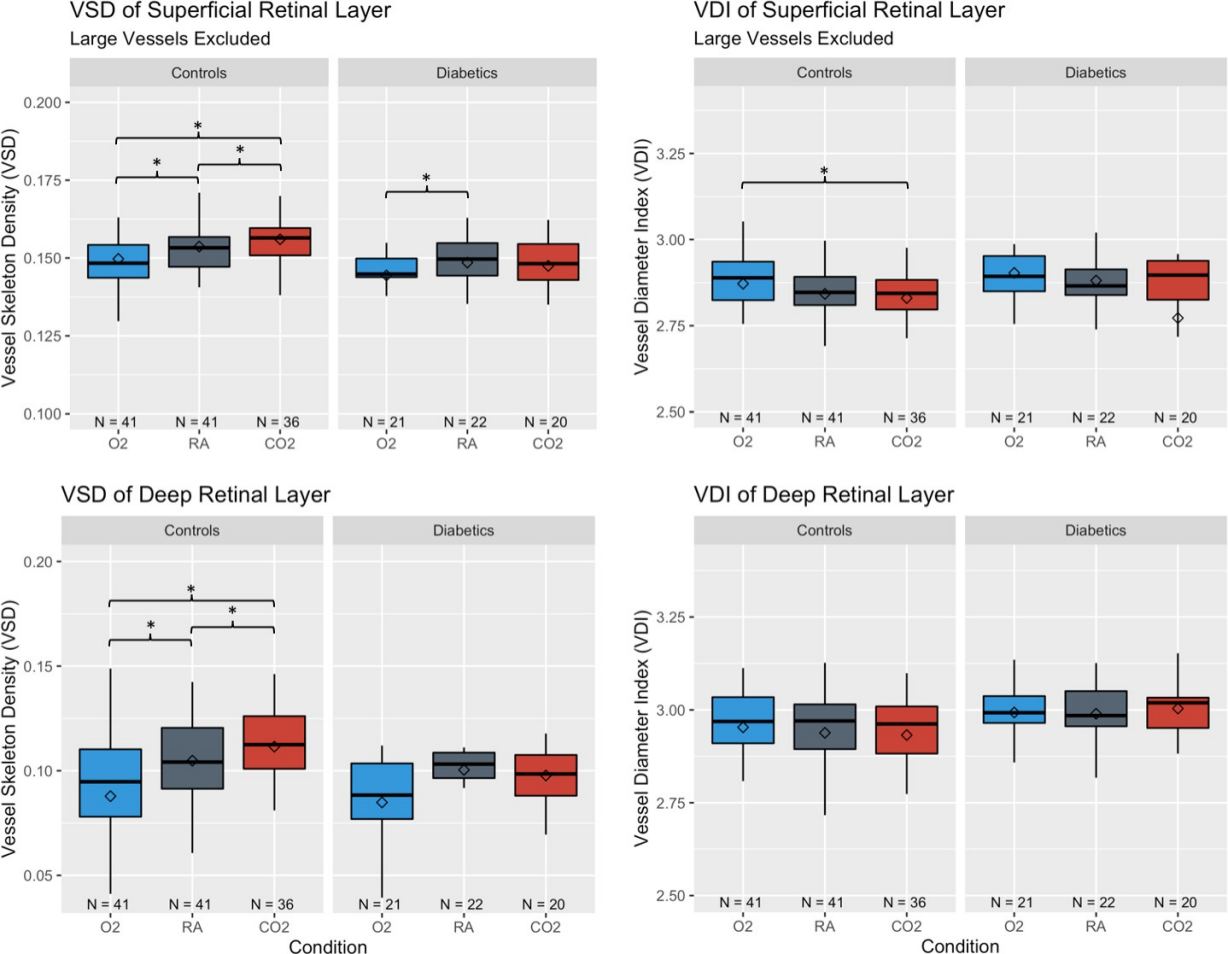
Vessel skeleton density (VSD) and vessel diameter index (VDI) of control and diabetic subjects under gas conditions. *Whiskers* indicate highest or lowest point within 1.5 times the interquartile range from the upper or lower quartile. *Diamonds* indicate the mean. *Stars* indicate significance for pairwise comparison between conditions from the ANCOVA model based on Bonferroni adjusted p-value of 0.017.

There was no significant difference in mean VSD of diabetic subjects for the pairwise comparisons between room air and CO_2_ gas conditions (SRL_RA_ 0.148 ± 0.002 vs SRL_CO2_ 0.146 ± 0.002, p = 0.072). As Fig 4 shows, the reactivity of retinal capillaries to CO_2_ was not only absent in the SRL of diabetic subjects but the mean VSD under CO_2_ was paradoxically lower than RA. In contrast, in the SRL of control subjects, there was a robust difference in all pairwise comparisons of the mean VSD among the three gas conditions in the expected directions (SRL_RA_ 0.154 ± 0.001 vs SRL_CO2_ 0.156 ± 0.002 vs SRL_O2_ 0.150 ± 0.002, p<0.017). Results for VAD were consistent with those of VSD, and the VSD results were unchanged when large vessels were not excluded from the SRL (see S1 Fig and S2 Fig). The omnibus findings of the mixed factorial analysis confirmed a significant gas condition (p<0.001), layer (p<0.001) and gas-layer interaction (p = 0.001) in the controls. In diabetic subjects, the mixed factorial analysis confirmed that there was a significant effect of gas condition (p = 0.005), mainly driven by the response to O_2_, layer (p<0.001) as well as a significant gas-layer interaction (p = 0.02).

For the VDI metrics, there was a significant gas effect in the SRL of control subjects (SRL_RA_ 2.84 ± 0.01 vs SRL_CO2_ 2.83 ± 0.01 vs SRL_O2_ 2.87 ± 0.02, p = 0.001) which was driven primarily by the difference in VDI between O_2_ and CO_2_ (p = 0.002). Gas effect on the VDI metric in the SRL was not present in diabetic subjects (SRL_RA_ 2.88 ± 0.03 vs SRL_CO2_ 2.77 ± 0.03 vs SRL_O2_ 2.90 ± 0.02, p = 0.44). In the controls, the mixed factorial analysis confirmed that there was a significant effect of both gas conditions (p = 0.006) and layer (p<0.001) on VDI but no significant gas-layer interaction (p = 0.29). In diabetic subjects, the mixed factorial analysis neither confirmed a significant effect of gas condition (p = 0.42) nor a significant gas-layer effect (p = 0.34) but did show a significant difference between layers (p = 0.009). The VDI results were similar when large vessels were not excluded from the SRL (see S2 Fig).

Unlike the controls with significant pairwise comparisons between gas conditions (p<0.017), all pairwise comparisons of VSD between gas conditions in the DRL of diabetic subjects were non-significant. The mixed factorial analysis of the gas effect in the diabetics however showed significance (p = 0.005) and appeared to be driven by the response to hyperoxia from room air in the SRL (p = 0.005). The VSD response to hyperoxia in the DRL was trending towards significance (p = 0.019) but did not reach significance with the Bonferroni correction. Notably, as in the SRL, the difference in the VSD of the DRL under hypercapnic conditions was in a paradoxical direction and may reflect an aberrant physiological response in the diabetic subjects. In the controls the mixed factorial analysis confirmed significant gas effects on VSD in the DRL (DRL_RA_ 0.105 ± 0.003, DRL_CO2_ 0.112 ± 0.003, DRL_O2_ 0.087 ± 0.006, p<0.001). Results for VAD in the DRL were consistent with those of VSD (see S1 Fig). Neither the controls nor subjects with diabetes had a significant gas effect on the VDI within the DRL.

## Discussion

We investigated layer specific RVR during physiologically salient changes in inhaled oxygen and carbon dioxide in diabetic subjects and non-diabetic controls using SS-OCTA. Consistent with our previous findings [19] we found retinal vascular reactivity impairment in diabetic subjects can be assessed with OCTA and show that even in the early stages of the disease the impairment to hypercapnia previously reported are present. Whereas our earlier study consisted entirely of DR subjects with the full range of disease severity, our current cohort of diabetic subjects included only those without DR (68%) or those with mild NPDR. We extend our previous findings by demonstrating that the impairment in RVR in diabetic subjects is present in both the SRL and DRL and is more profound in the DRL. Namely, there was no significant effect of any gas condition on apparent capillary density in the DRL among diabetic subjects by pairwise comparison. Lastly, we show that the RVR findings are present even when larger arterioles and venules are excluded from the images. This suggests that capillaries themselves mediate the RVR and that this physiological response is abnormal in DR.

We found that retinal vascular reactivity to hyperoxia was preserved in the SRL of diabetic subjects but not significant in the DRL, even in the early stages of the disease. This is consistent with the recent report by Meshi et. al. that among diabetic subjects without DR, capillary density in the deep capillary plexus but not the superficial capillary plexus was significantly lower than that of controls [24]. Various other studies have reported a similar result for type 1 diabetics with no or mild DR [21–23]. It may therefore be that capillary damage in the DRL, including the ability to autoregulate, is more extensive than damage to the SRL in early disease.

We primarily used a measure of capillary density, VSD, which represents the length of capillaries in the image. Changes to VSD with the gas conditions may reflect blood flow within previously unperfused capillaries or vice versa, but more likely reflects incremental changes in flow through capillary segments. Due to the detection sensitivity of OCTA imaging in which flow below a certain threshold cannot be resolved, loss of signal for a given capillary segment between gas conditions may be from decreased perfusion through that segment outside the detection limits of the device. Differences in the VSD measures under the different gas conditions are thus convincing evidence of the change in perfusion per unit area.

Since capillaries typically only pass individual erythrocytes, it is possible that they function in binary states (only have open and closed states). Although it is not clear if Poiseuille's relation between flow rate and vessel diameter applies to capillaries, the changes in perfusion measures captured in our VSD metric may also be due to changes of vessel caliber induced by the gas conditions. The VDI results in the SRL were lower with increased CO_2_ content in the controls. As VDI is a ratio of total vessel area (VAD) divided by skeletonized vessel length (VSD), the low VDI observed in the carbon dioxide condition compared to the oxygen condition is likely driven by a relatively large increase in vessel length compared to vessel width. Hence, the changes in VAD under the gas perturbation conditions are largely driven by the absence or presence of flow segments, rather than changes to flow caliber at the capillary level. This may suggest that capillary blood flow changes to gas perturbation function in a more binary manner (open or closed). A similar VDI trend was not noticed in the DRL of the controls and could mean the vessel dilation was simply neutralized by an increase in appearance of new vessel segments. The lack of changing VDI in the DRL could also be due to the confounding effect of OCTA signal [35].

Capillary pericyte loss, endothelial cells depletion and basement membrane thickening are among the earliest vasobliterations associated with clinically detectible signs diabetic retinopathy [35, 36]. The potential mechanisms underlying retinal vascular reactivity assessment at the capillary level, such as assessed by our study, provide a pathway for implementing a clinically feasible protocol that can be an early biomarker of vascular function. Future studies may explore retinal vascular reactivity measures at the capillary level as a potential clinical variable for investigating the wide variability in the duration between when a subject first develops diabetes to when retinopathy is diagnosed.

The findings of our study come with some limitations. Namely our sample size was small and therefore we were unable to control for all potential confounders of retinal vascular reactivity including smoking [37]. Even though our sample size was small, our data was representative of the general trends previously described by our group and others, namely, that the mean VSD for the diabetic subjects was lower than the controls and VSD in the SRL was consistently greater than in the DRL. In addition, the relationship of VSD among retinal layers was predictable among controls and agreed with previous findings. One other limitation of our study is that the gas conditions we used were relatively mild to ensure subject comfort and safety. Nevertheless, the methodology is very useful because it can be applied using commercially available devices and on living human subjects with reasonable and reliable effect sizes. We have provided a detailed description of the methodology and its strengths and limitations in our previous publication Ashimatey et al. [19] as well as in Kushner-Lenhoff et al. [25]

## Supporting information

S1 Fig Vessel area density (VAD) of control and diabetic subjects under gas conditions. *Whiskers* indicate highest or lowest point within 1.5 times the interquartile range from the upper or lower quartile. *Diamonds* indicate the mean. *Stars* indicate significance for pairwise comparison between conditions from the ANCOVA model based on Bonferroni adjusted p-value of 0.017.
(TIF)

S2 Fig Vessel skeleton density (VSD) and vessel diameter index (VDI) of control and diabetic subjects under gas conditions using all vessel analysis in the superficial retinal layer. *Whiskers* indicate highest or lowest point within 1.5 times the interquartile range from the upper or lower quartile. *Diamonds* indicate the mean. *Stars* indicate significance for pairwise comparison between conditions from the ANCOVA model based on Bonferroni adjusted p-value of 0.017.
(TIF)
