# Supplementary figures and images for: Impaired layer specific retinal vascular reactivity among diabetic subjects
Source: PLoS One. 2020 Sep 11;15(9):e0233871. doi: 10.1371/journal.pone.0233871 (PMC7485884; doi:10.1371/journal.pone.0233871)

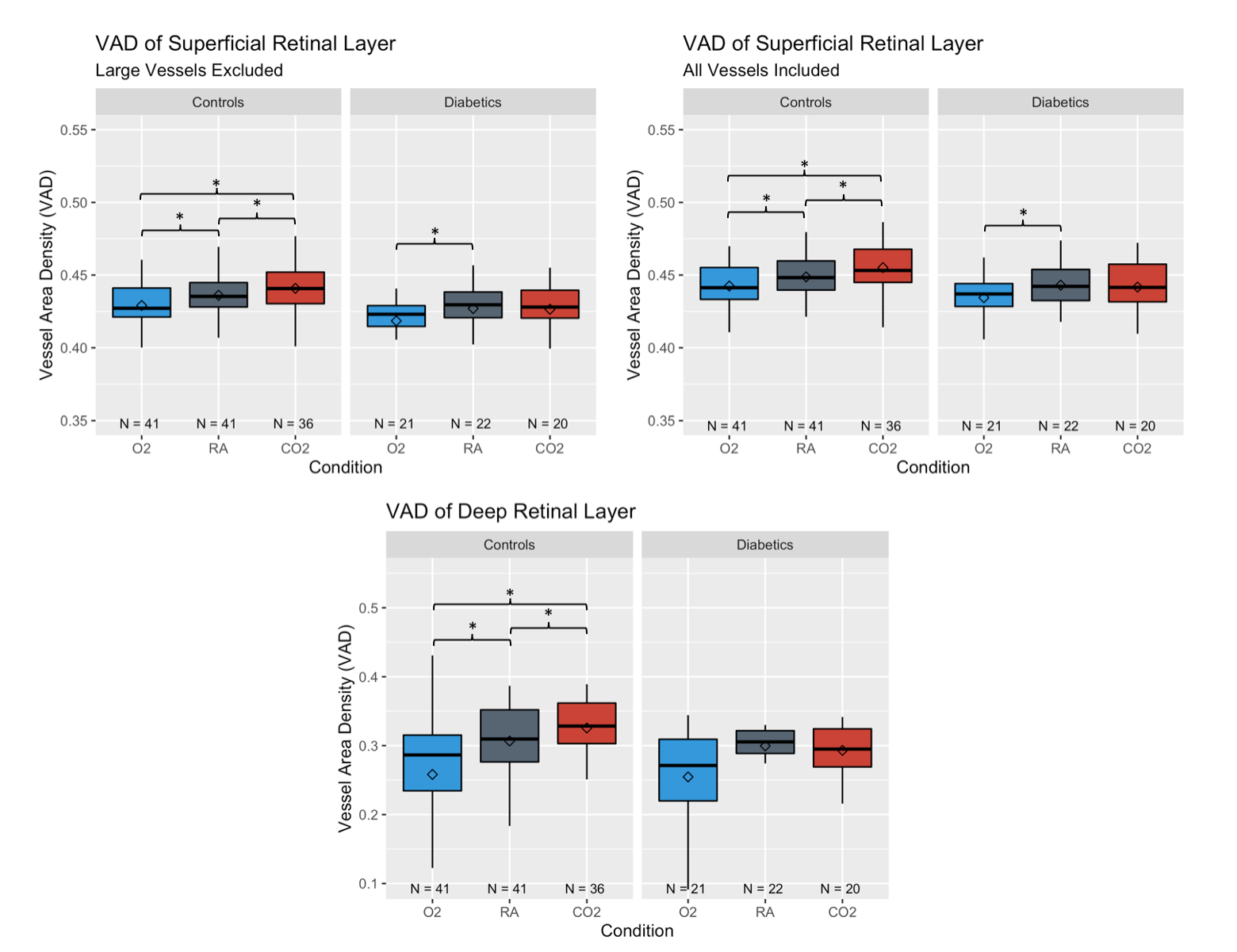

Supplement: S1 Fig — Whiskers indicate highest or lowest point within 1.5 times the interquartile range from the upper or lower quartile. Diamonds indicate the mean. Stars indicate significance for pairwise comparison between conditions from the ANCOVA model based on Bonferroni adjusted p-value of 0.017. (TIF) [file pone.0233871.s001.tif]

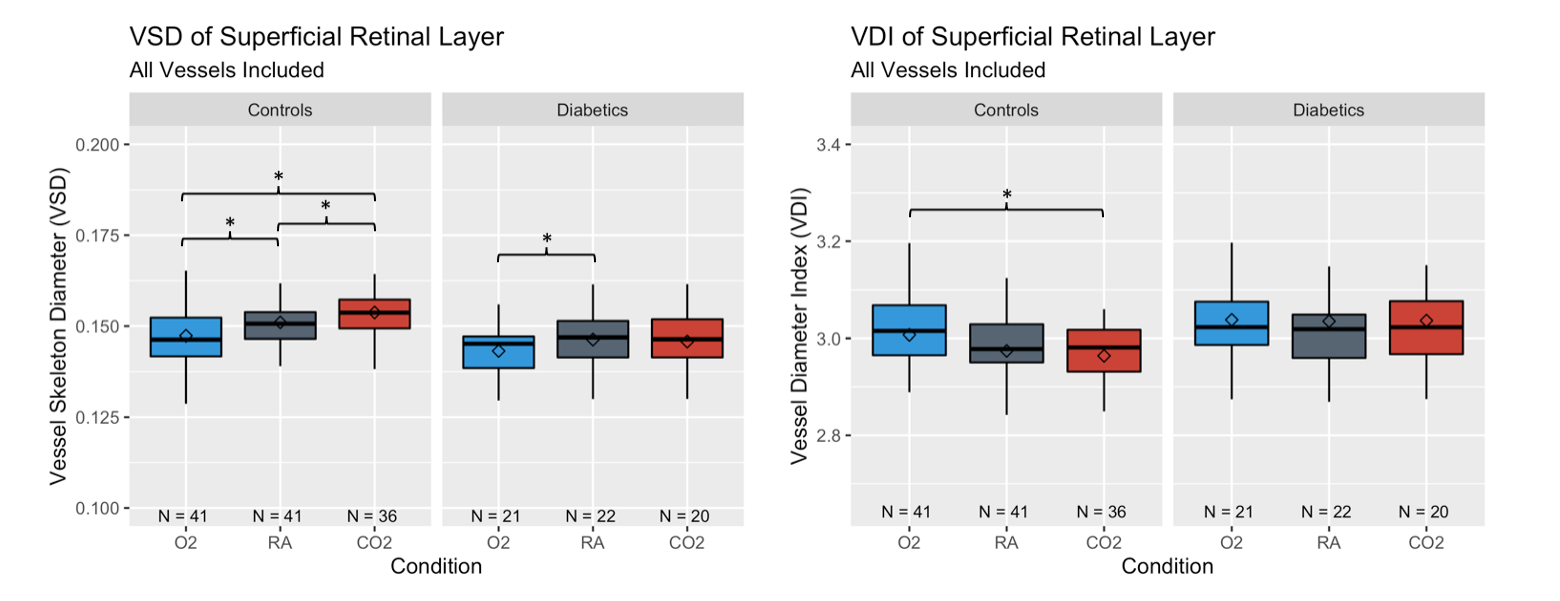

Supplement: S2 Fig — Whiskers indicate highest or lowest point within 1.5 times the interquartile range from the upper or lower quartile. Diamonds indicate the mean. Stars indicate significance for pairwise comparison between conditions from the ANCOVA model based on Bonferroni adjusted p-value of 0.017. (TIF) [file pone.0233871.s002.tif]
